# Influences of Increased Pressure Foaming on the Cellular Structure and Compressive Properties of In Situ Al-4.5%Cu-xTiB_2_ Composite Foams with Different Particle Fraction

**DOI:** 10.3390/ma14102612

**Published:** 2021-05-17

**Authors:** Zhengyi Niu, Zhentao An, Zhibao Jiang, Zhuokun Cao, Yang Yu

**Affiliations:** 1 Department of Ammunition Engineering, Army Engineering University of PLA, Shijiazhuang 050003, China; ideal258@163.com; 2 32181 Unit of PLA, Shijiazhuang 050003, China; czkstick@163.com; 3 School of Metallurgy, Northeastern University, Shenyang 110089, China; 1610507@stu.neu.edu.cn

**Keywords:** metallic foams, in situ particles, increased pressure foaming, energy absorption

## Abstract

Metallic foams have drawn increasing attention in applications ranging from lightweight structures to energy absorption devices. Mechanical properties of metallic foams depend on both their microstructure and cellular structure. In situ Al-4.5%Cu-xTiB_2_ composites were used as start materials for fabrication of closed-cell foams through liquid route under atmosphere pressure and increased pressure, aiming at simultaneously strengthening the cell wall material and optimizing the cellular structure. Macro-structural features of the foams were determined by micro X-ray computed tomography (µCT); results exhibit that increasing weight ratio of in situ TiB_2_ particles leads to coarsened cell structure for foams made under atmosphere pressure, due to the increase in critical thickness of cell wall rupture. Significant reduction of cell size and increase in cell circularity were observed for foams fabricated under increased pressure. Quasi static compression test results indicate that yield strength of foam samples increases with increasing particle fraction and refinement of cell structure. Microstructure observation shows that the continuous network at interdendritic regions consists of in situ TiB_2_ particles and intermetallic compounds are responsible for the reduced ductility of cell wall materials and the reduction in energy absorption efficiency of foams with high particle fraction. The influences of cell structure on the normalized strength and specific energy absorption were also discussed, and it was found that the improvement of yield strength and energy absorption of composite foams attributes to both the reinforcement of in situ TiB_2_ particles and the refinement of cellular structure.

## 1. Introduction

Metallic foams have received much attention for automobile, aerospace, and structural applications due to their unique combination of light weight, high specific strength and specific stiffness, as well as energy absorption capacity and damping ability [1,2,3]. Mechanical properties of closed-cell metallic foams depend on both the microstructure of cell wall material and the geometric structure of cells, so there is scientific interest in finding ways to improve the mechanical properties of cell wall material and optimize the cellular structure at the same time.

In the past decades, different types of processing routes have been developed to manufacture closed-cell aluminum foams. In liquid foaming route or powder metallurgy foaming processes, enhancement of liquid foam stability has been reported by introducing alloying elements Ca or Mg [4,5] as well as ex situ particles such as SiC and Al_2_O_3_ with appropriate size and volume fraction [6,7]. Experimental results indicate that the addition of thickening agents or ceramic particles into aluminum matrix leads to a notable influence on the cell structure and mechanical properties of aluminum-based foams [8,9,10].

Compared to ex situ, in situ TiB_2_ particles reinforced aluminum matrix composites exhibit superior mechanical properties for the small particle size and strong interface bonding [11]. Nevertheless, in situ TiB_2_ particles do not attach to the gas–liquid interface during the foaming process for their good wettability with molten aluminum and their influence on liquid foam stability and mechanical properties of composite foams are not clear yet [12,13]. Kennedy reported an increase in foam expansion and improvement in compressive strength of aluminum foams prepared by applying powder metallurgy route with ex situ TiB_2_ particles addition [14]. Heim’s work shows that in situ TiB_2_-reinforced aluminum matrix composites are stability foamable when applying a gas injection foaming technique [15]. However, Athul Atturan et al. found that increasing in situ TiB_2_ particles fraction would lead to more coalescence in liquid aluminum foam at same holding time [16]. The lack of foam stability and inhomogeneity in cellular structure leads to decrease of compressive strength and energy absorption of A357 alloy-based composite foams when the weight fraction of in situ TiB_2_ particles increased from 5.0% to 10.0% [17].

Varying TiB_2_ fraction is reported to cause a significant improvement in mechanical properties of in situ Al-4.5%Cu-xTiB_2_ composites [18,19]. In the present work, aiming at simultaneously optimizing the microstructure and cellular structure of composites foams, metallic Ca was added to molten Al-4.5%Cu-xTiB_2_ composites to improve the stability of liquid foams, and increased pressure foaming was preformed to modify the cellular structure. The effects of varying TiB_2_ fraction and changing in cellular structure on the mechanical properties of composite foams were investigated.

## 2. Materials and Methods

### 2.1. Composites Fabrication

In situ Al-TiB_2_ composites were prepared by mixing K_2_TiF_6_, KBF_4_ and Na_3_AlF_6_ salts into molten aluminum at 850 °C. The salt–metal mixture was stirred under electromagnetic stirring at a frequency of 25 Hz for 30 min. The chemical composition of fabricated in situ Al-TiB_2_ composites is listed in Table 1, and the weight fraction of in situ TiB_2_ particles is estimated to be 10.2 wt.%.

### 2.2. Increased Pressure Foaming

Composites was melted in a steel crucible; the weight fraction and of in situ TiB_2_ particles and Cu were adjusted with mixing commercial pure aluminum and Al-50Cu master alloy into molten Al-TiB_2_ composites. Metallic Ca was used as thickening agents, which follows the Alporas process [4]. After 2.5 wt.% Ca was added to the melt at 720 °C, the melt was stirred by a graphite impeller for 300 s.

The foaming process took place in a bottom sealed stainless tube at 690 °C. TiH_2_ particles, 1.2% in weight, were added to molted composite under mechanical stirring speed of 1200 rad/min for 180 s. The upper cover of the stainless tube was sealed as soon as the stirring process had finished, and the pressure inside the sealed device was kept at 0.24 MPa by Ar gas. After the foaming process was finished, the whole device was kept sealed, pulled out of the furnace and cooled by air, and the gas pressure inside the tube was kept unchanged until total solidification of the liquid foam. A detailed foaming device and procedure can be found elsewhere [20]. For comparison, foam samples were also prepared under atmosphere pressure applying the same procedure.

### 2.3. Structural Characterization

Density of aluminum foams ρ* was determined by measuring the weight of cubic foam specimens cut by wire electrical discharge machining (WEDM). X-ray tomography was performed on foam specimens using a Hamamatsu L9421-02 microfocus X-ray source manufactured by Hamamatsu Photonics K.K, Iwata, Japan and a flat panel detector. After reconstruction, the commercial software VGStudio Max 3.4 provided by Volume Graphics (Beijing) Technology Co., Ltd. China, was used to extract 2D slices for determination of cell size and circularity using software Imagepro Plus 6.0 provided by Media Cybernetics in Rockville, MD, USA. Cell size D was represented as an equivalent diameter D of a circle with the same area. Mean cell size D_m_ was determined by the cell size distributions based on area fraction. Area fraction is defined as the area contribution of a certain cell size class compared with the total area of all the cells. The circularity C of a cell is defined as 4πA/P^2^, where A and P are the area and perimeter of that cell, and the mean circularity C_m_ of the cells was determined by calculating the arithmetic average of all cells. A JSM-IT500 scanning electron micrography manufactured by JEOL Ltd., Tokyo, Japan was used to observe the micro structure of foam specimens. The cell wall thickness t was measured by a tool under a Nikon LV150N optical microscope assembled by Nikon Instruments Shanghai Co. Ltd. China. The apparent thickness of a cell wall was estimated by three measurements at 1/4, 1/2 and 3/4 of the cell wall length.

### 2.4. Compression Tests

Cubic compression specimens were cut using WEDM with 30 mm long sides. Quasi static compression tests were carried out on Shimadazu AG-X plus 100 kN universal testing machine manufactured by Shimadazu Corporation, Kyoto, Japan and the cross-head speed was 2 mm/min.

## 3. Results

### 3.1. Cellular Structure

**Figure 1:**
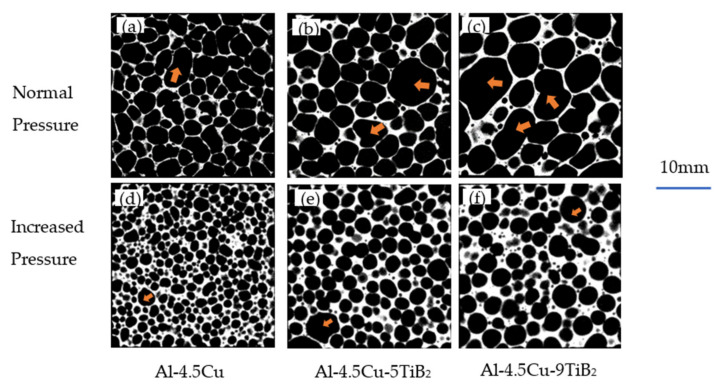
Cellular structure of foam specimens fabricated under atmosphere pressure: (**a**) Al-4.5Cu; (**b**) Al-4.5Cu-5TiB_2_; (**c**) Al-4.5Cu-9TiB_2_; and under increased pressure: (**d**) Al-4.5Cu; (**e**) Al-4.5Cu-5TiB_2_; (**f**) Al-4.5Cu-9TiB_2._

Density and structural parameters of foam specimens with different TiB_2_ fraction and foaming conditions are listed in Table 2, and images of µCT slices of foam specimens are shown in Figure 1. It is evident that the macrocellular structure of composite foams has scientifically changed with increasing particle fraction and foaming under increased pressure.

When foaming under normal pressure, Al-4.5Cu foam shows isotropic and homogeneous cellular structure similar to the macro structure of Alporas type foams that are also produced with Ca addition [10]. The composite foams in contrast were found to have an apparent coarse cell structure with broad size distribution. There are more structurally weak cells with large cell size and non-circularity as the fraction of in situ TiB_2_ particles increases from 5% to 9%. A similar observation was reported by Athul Atturan et al.—in A357 reinforced with in situ TiB_2_ composite foams, bubble coalescence is more in case of 10%TiB_2_ foam compared to that of 5%TiB_2_ foam [16]. Vinod Kumar et al. observed single melt films with different volume fraction of in situ TiB_2_ particles, and found that TiB_2_ particles form clusters to stabilize liquid film, instead of single particles. The decrease in cluster size is believed to be responsible for the relatively thin cell walls with low TiB_2_ fraction [21]. Banhart’s investigation suggests that the stability of liquid metal foam is related to the existence of critical wall thickness, normally 30–180 µm, and the liquid film ruptures when the thickness below the critical value [22]. From the data listed in Table 2, a notable increase in cell wall thickness with increasing TiB_2_ fraction could be found, indicating that the large value of critical thickness of film rupture in composite foams with more TiB_2_ fraction is responsible for the increased bubble coalescence.

**Figure 2:**
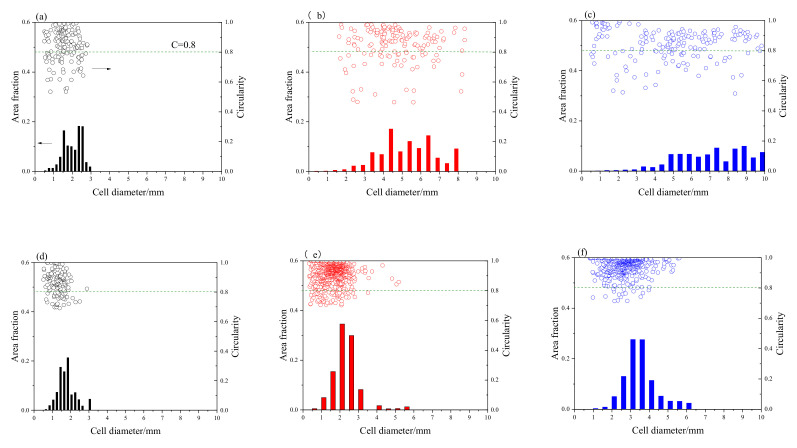
Cell size distribution and circularity of foam specimens fabricated under atmosphere pressure: (**a**) Al-4.5Cu; (**b**) Al-4.5Cu-5TiB_2_; (**c**) Al-4.5Cu-9TiB_2_; and under increased pressure: (**d**) Al-4.5Cu; (**e**) Al-4.5Cu-5TiB_2_; (**f**) Al-4.5Cu-9TiB_2._

By performing increased pressure foaming, it is clearly seen in Figure 2 that the cell size of foam specimens significantly decreases and the size distribution range narrows. It is worth noting that the correlation between mean cell size and density under increased pressure foaming is different compared to conventional producing routes. Körner’s research works on aluminum foams stabilized by oxide networks and particles indicates that there is a linear relationship between mean cell diameter and density, i.e., d_m_ ∝ 1/ρ* [23,24]. The mean cell size data of foam specimens foamed under increased pressure listed in Table 2 are apparently smaller than the prediction of the linear model based on the cell size of foams under normal pressure, indicating that bubble coalescence is effectively suppressed during increased pressure foaming. It is evident that the cell size of foams still increases with increasing particle fraction, but cells in composite foams are mostly spherical with a high circularity of 0.94 and 0.95. When compared to Al-4.5Cu foam, the structure of composite foams with in situ TiB_2_ is closer to a wet foam [25]. A few big cells are also shown in Figure 1e,f, but their shape is nearly spherical.

As described by Simancik et. al, expansion of powder compact precursor was apparently affected by ambient atmosphere [26]. The oxidation of surface layer of powder compacts could be reduced and resulted in a higher expansion when Ar was used as protecting gas. However, in the present work, Ar gas was only applied to increase the surrounding pressure. The oxidation of aluminum during the foaming process was not avoided, because the oxygen inside foaming tube was not removed before injection of Ar gas.

### 3.2. Compressive Properties

**Figure 3:**
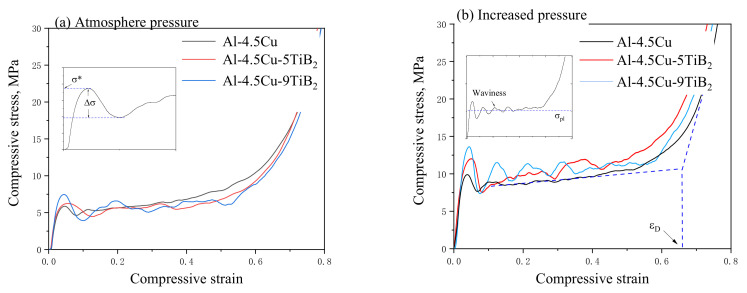
Compressive stress–strain curves of Al-4.5Cu-xTiB_2_ foams fabricated under (**a**) atmosphere pressure and (**b**) increased pressure.

To directly compare the compressive properties of composite foams with different in situ particles fraction, foam specimens with similar density were selected to perform quasistatic uniaxial compressive tests. Density and experimental data of specimens are listed in Table 3, and compressive stress–strain curves are shown in Figure 3.

When fabricated under atmosphere pressure, Al-4.5Cu foam shows a stress–strain curve of a typical low-density ductile foam, see Figure 3a. After the first elastic deformation region, a stress peak is observed, followed by a region of slight strain softening to a plateau, during which the stress remains nearly constant until densification. The peak stress at the end of the elastic region is selected as the yield strength σ* of a foam specimen. When the weight fraction of in situ TiB_2_ fraction increases from 0 to 5% and 9%, the peak stress increases from 5.8 MPa to 6.3 MPa and 7.4 MPa, or alternatively increases by 8.6% and 27.6%. This increase in compressive strength of foam specimen with increased in situ TiB_2_ fraction is in accordance with the strengthening effect of in situ TiB_2_ particles on tensile strength of dense Al-4.5Cu matrix composites [18]. Nevertheless, the stress drop Δσ after the stress peak and waviness in the plateau region increase for composite foams as the fraction of in situ TiB_2_ particles increases, which is typically observed in brittle foams [27].

As shown in Figure 3b, the compressive stress–strain curves for foams made under increased pressure are similar to that shown in Figure 3a, but the stress level is remarkably elevated. As the weight fraction of in situ TiB_2_ fraction increases to 5% and 9%, the peak stress increases 21.1% and 39.4% compared to that of Al-4.5Cu foam. The stress peak in the stress–strain curve corresponds to the onset of global collapse of cells, which is related to the mechanical properties of the cell wall and the cellular structure [28]. In the case of foams from the same starting material, structural parameters, such as cell size and circularity, are generally considered in most relevant studies. Consequently, large cells with low circularity are likely to undergo plastic collapse at lower loads [29]. Thus, the refinement of cellular structure when foaming under increased pressure is responsible for the remarkable increase in peak stress. Nevertheless, as shown in Table 3, the stress drop ratio Δσ/σ* for foams with same composition remains similar value, indicating the stress drop and waviness is mostly related to the ductile or brittle nature of the cell wall materials.

### 3.3. Deformation Behavior

**Figure 4:**
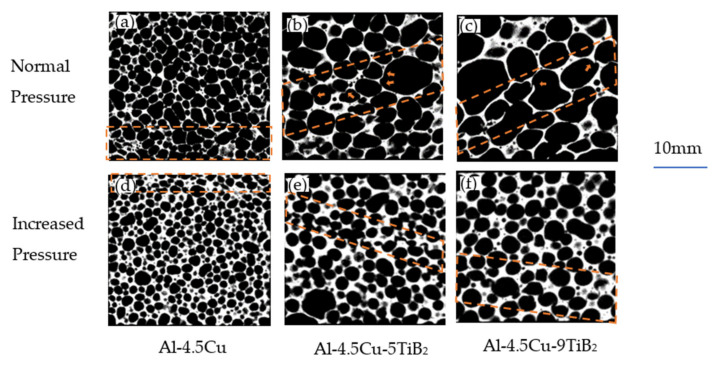
Deformation behavior at ε = 0.07 of foam specimens under atmosphere pressure: (**a**) Al-4.5Cu; (**b**) Al-4.5Cu-5TiB_2_; (**c**) Al-4.5Cu-9TiB_2_; and under increased pressure: (**d**) Al-4.5Cu; (**e**) Al-4.5Cu-5TiB_2_; (**f**) Al-4.5Cu-9TiB_2_.

To investigate the initial collapse of foam specimens, compressive tests were interrupted at strain ε = 0.07, corresponding to the first stress valley in the stress–strain curve of Al-4.5Cu foam in Figure 3a. Figure 4 shows the images of CT slices at the midplane of foam specimens. In Figure 4a, only a few deformed cells are visible in the bottom layer of cells, and the deformation is possibly distributed to multilayers because of its fine structure. In contrast, there are obvious cell wall bending and fracture adjacent to large or ellipse cells in Figure 4b,c for foams with in situ TiB_2_ particles. This confirms that large cells with low circularity play an important role in the initial collapse of Al-4.5Cu-xTiB_2_ foams. Fracture of straight cell wall without bending in Al-4.5Cu-9TiB_2_ foam specimen reveals the brittle nature of cell wall materials.

Due to the fine cell size, the strain corresponding to stress valley of foams fabricated under increased pressure is smaller than 7%, so a collapse band is already formed in Figure 4d–f. In Figure 4e,f, it is clearly seen that the position of the deformation band is not related to the cells with largest size or highest aspect ratio. One possible reason is that the size of these structural defects is relatively small compared to specimen size, so a single large cell or a missing cell wall is not able to cause the plastic collapse of the whole layer. Another reason is the fact that large cells in foam specimens fabricated under increased pressure are nearly spherical, which contributes to the reduction of stress concentration caused by cell structure inhomogeneity. In the collapse band of Al-4.5Cu foam, most cell walls are bent without fracture. Whereas, in Al-4.5Cu-5TiB_2_ foam, the bent cell walls are partly fractured, and most deformed cell walls are fractured in Al-4.5Cu-9TiB_2_ foam. These results also confirm that the obvious stress drop of composite foams is related to the brittleness of cell wall material when the weight fraction of in situ TiB_2_ particles increases.

**Figure 5:**
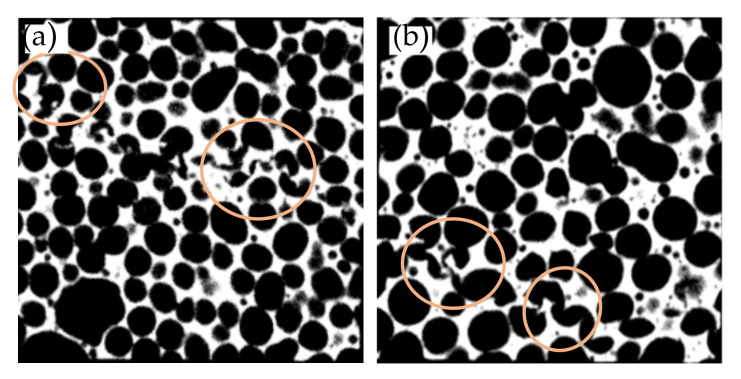
Deformation of composite foams processed under increased pressure at ε = 0.15 (**a**) Al-4.5Cu-5TiB_2_; (**b**) Al-4.5Cu-9TiB_2._3.4.

Images of CT slices of composite foams processed under increased pressure at ε = 0.15 are shown in Figure 5. It is observed that fractured cell walls come in contact with the neighboring cell walls. This interaction and slide of these fractured cell walls are responsible for the change in waviness and serrations in stress–strain curves.

Energy absorption of foam specimens calculated from stress–strain data are compared in Figure 4. Three foams fabricated under atmosphere pressure exhibit similar energy absorption values when the compressive strain is below 0.4. When the strain is over 0.4, Al-4.5Cu exhibits higher energy absorption per unit volume, which is related to the strain hardening observed in the plateau deformation region in stress–strain curves. For foam specimens made under increased pressure, composite foams with in situ TiB_2_ particles show higher energy absorption values than Al-4.5Cu foam, which contributes to the increased plateau stress.

Figure 4 also shows the energy absorption efficiency of the foam specimens during compression, which is defined as

(1) $$\eta =\frac{\int_{0}^{\varepsilon }\sigma \left(\varepsilon \right)d\varepsilon }{\sigma_{max}\left(\varepsilon \right)\varepsilon }$$

In which *σ*(*ε*) is the stress at strain *ε*, *σ_max_*(*ε*) is the maximum stress experienced by the foam up to the strain *ε*. For ideal plastic foams that exhibit constant plateau stress, *η* equals to 1, whereas *η* = 0.5 for elastic brittle foams [30].

As shown in Figure 4a, the energy absorption efficiency of Al-4.5Cu foam reaches a maximal value of 0.88 at ε = 0.3, and then gradually decreases to 0.6 at ε = 0.6. This value is similar to that for Alporas foam, for which η~0.9 and is recognized as a typical ductile foam [31].

For Al-4.5Cu-5TiB_2_ foam, η is over 80% in the plateau deformation region and the maximal value is 87%, attributed to the flat plateau stress. η drops approximately 10% to over 70% for Al-4.5Cu-9TiB_2_ foam at the plateau deformation region, showing the decrease in ductility of foam specimens when the weight fraction of in situ TiB_2_ particles increases to 9%.

**Figure 6:**
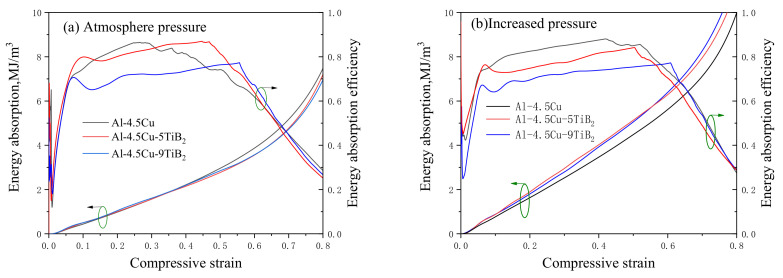
Energy absorption properties of Al-4.5Cu-xTiB_2_ foams.

In Figure 6b, η for Al-4.5Cu foam gradually increases during plateau region because of the weaker strain hardening at plateau deformation region compared to that of specimen made under normal pressure. There is a visible decrease of η for the Al-4.5Cu-5TiB_2_ foam specimen when foamed under increased pressure, corresponding to high stress drop ratio, as well as waviness and serrations in the stress–strain curve. The correlation between η and ε is quite similar for Al-4.5Cu-9TiB_2_ foam fabricated under different pressures, which is caused by the brittle nature of cell wall materials. It is also noted that the plateau region in η slopes is relatively long and smooth for foams made under increased pressure, which results from the lesser strain hardening during plateau deformation region. Strain hardening in metallic foams is related to the structural variability in cell size and density that causes the weakest struts to deform first, followed by the elastic loading of the sample until the second weakest cells, and so on [31]. In foams made under increased pressure, the cell size distribution range is narrow and the cell shape is normally an equiaxed sphere. As a result, the difference of strength between firstly deformed band and following collapse layers is reasonably small, which causes less stress hardening and results in a long plateau in η slopes.

### 3.4. Microstrucutre

**Figure 7:**
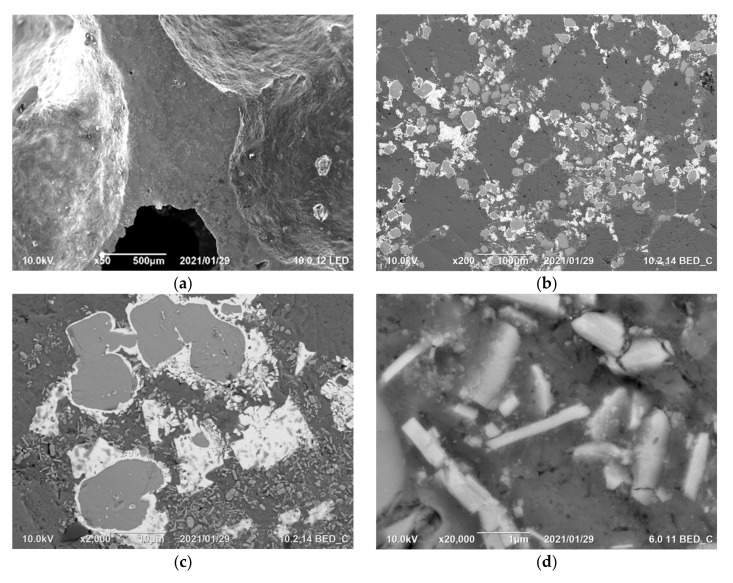
SEM micrography of a cell wall in Al-4.5Cu-9TiB_2_ foam fabricated under increased pressure: (**a**) cell wall, (**b**) microstructure of cell wall, (**c**) intermetallic compouds, (**d**) TiB_2_ particles.

The microstructure of Al-4.5Cu-9TiB_2_ foam fabricated under increased pressure was observed using scanning electron microscope. As shown in Figure 7, a continuous network consisting of fine particles and intermetallic compounds was observed at interdendritic regions of αAl. Such agglomeration of particles and second phases could make the cell walls very brittle in nature during compression. The size of in situ TiB_2_ particles is normally 0.5 to 2 µm, and they are embedded in an aluminum matrix with tight interface bonding. As discussed in a number of works, for the fine size and clear interface with aluminum matrix, ductility of in situ Al-Cu-xTiB_2_ would decrease when x > 5, caused by the accumulation of a thick layer of particles at the interdendritic region [18,19].

**Figure 8:**
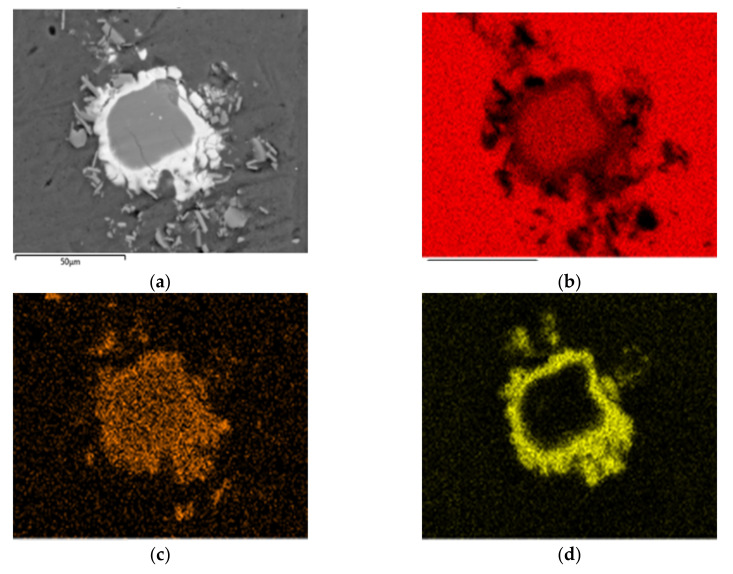
Element distribution of an intermetallic compound in Al-4.5Cu-9TiB_2_ Foam, (**a**) SEM micrograph, (**b**) Al, (**c**) Ca and (**d**) Cu.

Large quantities of intermetallic compounds also play an importance role in the reduction in ductility of cell wall materials. Huang et al. observed the microstructure of Al-Ca-Cu alloy with different Cu fraction, and their results also show that eutectic Al-Cu-Ca phases are with the volume fraction of 21.8% when the content of copper is only 5 wt.% [32]. In Figure 7b, it is seen that the intermetallic compounds are composed of two layers. Figure 8 shows the results of element distribution in a single compound. The inside phase contains mostly Al and Ca, while the outer layer has a high Cu and Ca concentration. However, no similar double layered phase was found in Huang’s work with varying Cu content. The formation of this unusual phase possibly results from the fact that the foaming procedure is quite different to the alloying technology.

## 4. Discussion

### 4.1. Stucture–Strength Correlation

The yield strength of dense in situ TiB_2_ reinforced aluminum alloy matrix composites is reported to show significant increase in tensile strength with increasing the fraction of in situ particles. In case of Al-4.5Cu-xTiB_2_ composites, the yield strength is reported to increase from 175 MPa to 208 MPa and 225 MPa when the weight fraction of TiB_2_ particles increases from 3.0% to 6.0% and 9.0% [18]. However, a decrease in compressive strength was reported when the weight fraction of in situ TiB_2_ particles in composite foams increased from 5% to 10 wt.%, which attributed to the increased number of structural weak cells with high aspect ratio and non-circularity [19]. In the present work, increasing the weight fraction of in situ TiB_2_ particles does lead to enlargement of cell size, but the average circularity of cells remains over 0.84, indicating that there is an improvement in foam stabilization with Ca addition. From the compressive test results, it is found that the yield strength σ* of foam specimens increases with increasing in situ particle fraction, which is in accordance with experimental results of dense composites. Thus, structural improvement is of crucial importance for metal matrix composite foams.

The correlation of yield strength σ* of foams, defined as the first peak stress after elastic deformation region, with the yield strength of cell wall material σ_s_ and relative density ρ*/ρ_s_ could be estimated by the equation derived by Gibson and Ashby as [28]:

(2) σ*σs=0.3φρ*ρs32+1−φρ*ρs

where *φ* is the fraction of solid that is contained in the cell edges. Respectively, yield strength of Al-4.5Cu alloy, Al-4.5Cu-5TiB_2_ and Al-4.5Cu-9TiB_2_ composites are taken as 148 MPa, 197 MPa and 225 MPa from literature [18,19]. That means the yield strength of composites increased by 40.7% and 52.0% compared to Al-4.5Cu alloy, when the weight fraction of in situ TiB_2_ particles increases to 5% and 9%.

As know from Equation (2), for foams with same relative density and similar cellular structure, the yield strength of foam specimens would increase linearly with increasing the yield strength of dense material. As mentioned above, the yield strength of composite foams fabricated under atmosphere pressure with 5% and 9% TiB_2_ particles increases only by 8.6% and 27.6% when compared to Al-4.5Cu foam, which is way lower than the increase in yield strength of dense material. Quite a lot of studies have compared experimental data of commercialized aluminum foams, such as Alporas, Cymat and Alulight, with Equation (2) with varied φ value. It was found that Equation (2) overestimates the strength, and most of experimental data are only comparable with open cell foams with φ = 1 [33,34]. It has been pointed out that defects such as partially coupled cells and missing cell walls can significantly reduce the plateau stress of closed-cell foams [35,36]. Thus, the main reason of the small increase in yield strength of composite foams fabricated under normal pressure is the initial collapsing of large and irregular pores at relatively low compressive stress.

When performing increased pressure foaming, the volume of gas bubbles is contracted and the size difference between neighboring bubbles reduces, so coalescing of bubbles during the foaming process is suppressed [20]. As shown in Figure 2, it is evident that cell size significantly decreases and mean cell circularity increases for all foam specimens processed under increased pressure. The refinement of cell structure with fine pores and round pores could result in homogeneity and better load distribution to avoid yield of foam specimen under a low stress. In the research work of Mukherjee et al., the structure and property correlation were discussed on three aluminum foams with different structural features, and the results indicate that decrease in cell size and increased circularity of large cells in foams could lead to improvement of mechanical performance [37]. As shown in Figure 2e,f, large cells in composite foams exhibit circularity value over 0.8, which is considered to eliminate stress concentration near large cells and avoid collapse under low strength. This result is also in accordance with the observation in Figure 4e,f that the formation of collapse bond is not related to the biggest cell in the cross-section. Due to the cellular structure refinement of foams processed under increased pressure, the yield strength of Al-4.5Cu-5TiB_2_ and Al-4.5Cu-9TiB_2_ composite foams increased by 21.1% and 39.4% compared to that of Al-4.5Cu foam. Nevertheless, even though structurally weak cells are rare in composite foams fabricated under increased pressure, their yield strength is still lower than the value predicted by Al-4.5Cu foam and the yield strength of dense material.

**Figure 9:**
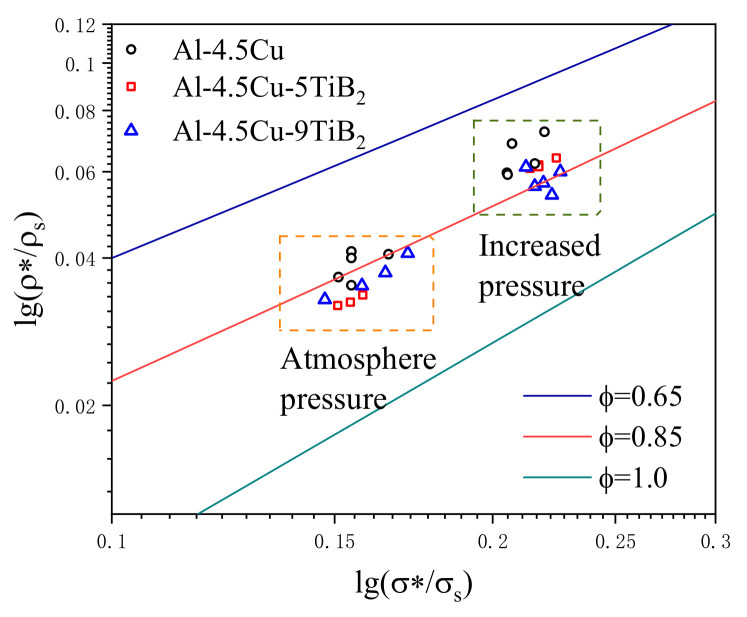
Comparison of experimental data with equations.

In Figure 9, experimental data are compared to Equation (2) with φ = 1, φ = 0.85 and φ = 0.65 [29]. Normalized stress of Al-4.5Cu alloy foam fabricated under increased pressure is between the two predicted lines representing φ = 0.65 and φ = 0.85. Whereas, composite foams are around the line of φ = 0.85. This indicates that a possible reason for the high normalized strength of Al-4.5Cu foam is related to a lower φ value, i.e., cell walls occupy more material. When compared to Al-4.5Cu foam, the structure of composite foams with in situ TiB_2_ is more similar to a wet foam [38]. The reason for this is that the viscosity of liquid melt significantly increases with the existence of in situ particles, leading to a reduced gravity drainage and causing a slow shrinkage of Plateau borders.

### 4.2. Specific Energy Absorption

**Figure 10:**
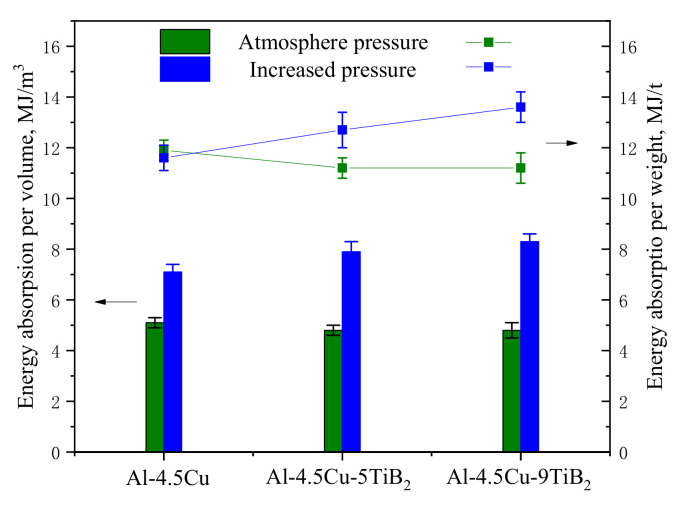
Energy absorption of foam specimens.

In most cases, metallic foams are used as energy absorption components. The energy absorption ability of metal foams is characterized by measuring the energy absorbed in crushing the material up to the densification strain ε_D_. Depending on the demand of application, specific energy absorption per unit volume or per unit weight are important aspects in evaluating the properties of metallic foams [39]. Data of specific energy absorption (SEA) till densification strain of all foam specimens are shown in Figure 10. SEA of foam specimens processed under atmosphere pressure show similar values, since foams with similar density show close values of densification strain. Whereas, composite foams with in situ TiB_2_ particles foamed under increased pressure show an increased capacity of energy absorption.

In the observation of in situ compression test under SEM, it is found that shear and friction of fractured cell walls provide additional energy absorption for brittle foams [40]. Images of CT slices of composite foams at ε = 0.15 are shown in Figure 5; it is seen that fractured cell walls in deformation band contact with the cell walls adjacent to the collapse layer, leading to the rise of stress, and provide additional energy dissipation. In contrast, there is little additional energy absorption in composite foams made under atmosphere pressure, because the cells are too large for fractured cell walls to interact with neighboring cells. Therefore, the decrease in cell size and porosity is the main reason of elevated specific energy absorption per unit weight for composite foams prepared under increased pressure.

## 5. Conclusions

In situ Al-4.5%Cu-xTiB_2_ composite foams were prepared under different pressures with Ca addition. Increase of particle fraction leads to coarsened cell structure of foam specimens under atmosphere pressure, due to the increase in critical thickness of cell wall rupture. The cellular structure of foam specimens is significantly refined when foaming under increased pressure, characterized by the fine cell size and high average circularity. Quasi static compression test results indicate that yield strength of composite foams increases with increasing particle fraction and refinement of cell structure. In situ TiB_2_ particles and intermetallic compounds accumulating at the interdendritic regions inside cell walls are the main reason of the reduced ductility of foams and the reduction of energy absorption efficiency. It is found that the significant improvement of yield strength and energy absorption of composite foams fabricated under increased pressure attributes to both the reinforcing effect of in situ TiB_2_ particles and the refinement of cellular structure.

## Figures and Tables

**Table 1:** Chemical compositions of in situ TiB_2_-reinforced aluminum matrix composites.

Elements	Ti	B	Si	Fe	V	Al
Weight %	6.985	3.217	0.085	0.154	0.006	Bal.

**Table 2:** Composition, density and structural parameters of foam specimens.

Foam Specimen	Applied Pressure MPa	ρ* gcm^−3^	d_m_ mm	t_m_ µm	C_m_
Al-4.5Cu	0.1	0.43 ± 0.02	2.9	52.1	0.87
Al-4.5Cu-5TiB_2_	0.1	0.44 ± 0.03	4.6	78.9	0.89
Al-4.5Cu-9TiB_2_	0.1	0.46 ± 0.04	6.7	126.8	0.85
Al-4.5Cu	0.24	0.58 ± 0.03	1.8	99.6	0.89
Al-4.5Cu-5TiB_2_	0.24	0.62 ± 0.02	2.3	119.6	0.92
Al-4.5Cu-9TiB_2_	0.24	0.64 ± 0.02	2.8	162.8	0.95

**Table 3:** Compressive properties of foam specimens.

Foam Specimen	Applied Pressure MPa	ρ* gcm^−3^	σ* MPa	Δσ MPa	Δσ/σ* %	σ_pl_ MPa
Al-4.5Cu	0.1	0.43	5.8	1.2	20.7	5.8
Al-4.5Cu-5TiB_2_	0.1	0.43	6.3	1.9	30.0	5.7
Al-4.5Cu-9TiB_2_	0.1	0.43	7.4	3.5	47.3	5.7
Al-4.5Cu	0.24	0.61	9.9	2.3	23.2	8.9
Al-4.5Cu-5TiB_2_	0.24	0.61	12.0	3.4	36.7	9.8
Al-4.5Cu-9TiB_2_	0.24	0.62	13.8	6.2	45.0	10.0

## Data Availability

Data sharing not available.
